# 114 Virtual Reality Combined with Multimodal Pain Management Allows for Clinic Based LASER Burn Scar Remodeling

**DOI:** 10.1093/jbcr/iraf019.114

**Published:** 2025-04-01

**Authors:** Matthew Bozeman, Azzolini Anthony, Michelle Broers, Jodi Wojcik, Ryan Shapiro

**Affiliations:** University of Louisville Burn Center; University of Louisville Burn Center; University of Louisville Burn Center; University of Louisville Burn Center; University of Louisville Burn Center

## Abstract

**Introduction:**

Hypertrophic scarring is noted to develop in about 70% of burn survivors with deep partial and full thickness burn injuries. Treatment of these scars is increasingly being performed with laser therapy. Pain control, patient comfort and facility preference have previously limited laser use in the literature to being performed in the operating room with sedation. In our experience, patients were often hesitant to proceed back to the OR for further therapy. Our hypothesis was same day clinic-based laser therapy would be possible with appropriate pain medication and VR headset-based distraction.

**Methods:**

Patients were seen at follow up and screened for either clinic or operating room-based fractionated CO2 laser therapy. Clinic patients received diazepam, oxycodone and topical analgesics with laser therapy performed after 30 minutes. Real-I Series VR headsets were utilized, one session with and one without and scores compared. Operating room-based therapy was performed with greater than 50% TBSA burns, per patient request, and on sensitive areas requiring monitored sedation. Self-reported pain scoring (1-10 scale), surface area treated, and acute stress and anxiety scores were recorded before and after treatments.

**Results:**

From 2022-2024, 225 separate laser therapy sessions were identified. Of these, 215 (90%) were able to be performed in clinic. We utilized the operating room for 25 (10%) treatments. Post procedurally, pain score was 5 (range 3-7) for clinic patients, which was not different than those performed in the operating room (4.9 range 2-7). Patients using VR headsets (8 total) had improvement in pain scores (7 without vs 5.1 with) and all chose to use the headsets at future appointments as a distraction technique. 2 patients crossed over to requiring the operating room for their repeat laser therapy sessions. The treated surface area ranged from 50sq cm to 1300 sq cm (1%-65% TBSA). Acute stress and anxiety scores were not negatively impacted with clinic-based laser treatment.

**Conclusions:**

VR headset use improves patient subjective pain scores during laser burn scar remodeling. Multimodal pain control techniques combined with VR headsets allow 90% of patients to have their procedures done same day in clinic, rather than utilizing the operating room.

**Applicability of Research to Practice:**

CO2 fractionated laser use is widespread and accepted for burn scar remodeling, and our technique allows for faster throughput, less use of the operating room, and theoretically lower costs without its use.

**Funding for the Study:**

N/A

